# Items

**Published:** 1903-12

**Authors:** 


### ITEMS.

MESSRS. SCHIEFFELIN & CO., New York, have placed us under obligations for a package of very fine flat pocket pencils, with point and eraser protected, for distribution to the editorial staff of the JOURNAL. One of the uses of the pencils suggested by the donors is to write prescriptions for hemoquinine. In case the point should be worn out in this process an extra tip is supplied for other uses.

---

THE DAILY MEDICAL JOURNAL will be published January 1, 1904. We need a physician as staff correspondent in every town in this state, to supply us with scientific, social, institutional and personal news, and will pay regular newspaper rates for this service. Instructions, stationery, and badge free; address Mr. J. Antchowvitsch, 15 East 72d Street, New York City.

